# Exploring Consumer and Patient Knowledge, Behavior, and Attitude Toward Medicinal and Lifestyle Products Purchased From the Internet: A Web-Based Survey

**DOI:** 10.2196/publichealth.5390

**Published:** 2016-07-18

**Authors:** Sulaf Assi, Jordan Thomas, Mohamed Haffar, David Osselton

**Affiliations:** ^1^ Faculty of Science and Technology Department of Archaeology, Anthropology and Forensic Science Bournemouth University Poole United Kingdom; ^2^ Faculty of Management Bournemouth University Poole United Kingdom

**Keywords:** consumer knowledge, attitude, behaviour authenticity, effectiveness, toxicity, counterfeit, medicinal and lifestyle products, Internet

## Abstract

**Background:**

In recent years, lifestyle products have emerged to help improve people’s physical and mental performance. The Internet plays a major role in the spread of these products. However, the literature has reported issues regarding the authenticity of medicines purchased from the Internet and the impact of counterfeit medicines on public health. Little or no data are available on the authenticity of lifestyle products and actual toxicity associated with their use and misuse.

**Objective:**

Our aim was to investigate consumer and patient attitudes toward the purchase of lifestyle products from the Internet, their knowledge of product authenticity and toxicity, and their experiences with counterfeit lifestyle products.

**Methods:**

A Web-based study was performed between May 2014 and May 2015. Uniform collection of data was performed through an anonymous online questionnaire. Participants were invited worldwide via email, social media, or personal communication to complete the online questionnaire. A total of 320 participants completed the questionnaire.

**Results:**

The results of the questionnaire showed that 208 (65.0%) participants purchased lifestyle products from the Internet mainly due to convenience and reduced cost. More than half (55.6%, 178/320) of participants purchased cosmetic products, whereas only a minority purchased medicinal products. Yet, 62.8% (201/320) of participants were aware of the presence of counterfeit lifestyle products from the Internet, and 11.9% (38/320) experienced counterfeit products. In only 0.9% (3/320) of those cases were counterfeit lifestyle products reported to authorities. Moreover, 7.2% (23/320) of the participants experienced adverse effects due to counterfeit lifestyle products.

**Conclusions:**

In summary, patients experienced counterfeit lifestyle products that resulted in adverse effects on their health. Although certain adverse effects were reported in this study, counterfeit products were underreported to authorities. Further public awareness campaigns and patient education are needed.

## Introduction

The last few years have witnessed a major change in perspectives toward medicine. Whereas medicine had been previously utilized for lifesaving purposes, a new era has emerged that involves the use of medicine to improve the overall lifestyle of individuals. In this respect, lifestyle products are intended mainly for improvement in mental functions and physical performance rather than curing diseases [1,2]. Lifestyle products can be of medicinal or non-medicinal origin, of any formulation type (eg, tablets, powders, creams, solutions), and any source (eg, herbal, synthetic). These products are classified into variable categories that improve mood and social behavior, cognitive functions, physical appearance, and/or sexual performance [1].

The Internet plays a major role in the dissemination of these products with many advantages over traditional marketplaces [3-6]. In this sense, the Internet offers a quick, easy, and more convenient way for purchasing medicinal and health care products. Specifically for medicinal products, the Internet offers regulated medicines without the need for a prescription [5,7]. Furthermore, Internet orders can be placed from home and at any hour of the day. Also, privacy is preserved with online purchases compared to face-to-face purchases [8], saving the consumer potential embarrassment. Additionally, the Internet provides more detailed information about the products and reduces visits to health care professionals and community pharmacies [7].

However, the issues associated with lifestyle products (including medicinal, herbal, dietary supplements, and cosmetics) purchased from the Internet are much more complicated. The purity and quality of these products represents a major concern and impact on consumer health [9-11]. For instance, these products may be defective in their packaging and ingredients. They may be poorly stored or past their best-before date. For instance, amphetamines and ephedrine encountered in counterfeit herbal weight loss products could result in sympathomimetic side effects such as hyperthermia, hypertension, and agitation [12,13]. Furthermore, heavy metals and pesticide contamination in counterfeit products often results in both acute and chronic side effects [14].

According to the World Health Organization, more than 50% of medicines purchased online could be counterfeit [15]. A further study conducted in the United States in 2004 showed that more than 80% of the medicines purchased from the Internet contained the wrong active pharmaceutical ingredient (API), were subpotent, out-of-date, or poorly stored [16]. Drug websites, unlicensed online pharmacies, and other unregulated online retailers have been found to sell counterfeit/substandard products [17,18]. In the United States, the Food and Drug Administration (FDA) warns against “rogue websites” that sell potentially dangerous drugs [19]. According to the FDA, these drugs may contain the wrong API, too much or too little API, or even dangerous API. Moreover, the National Association of Boards of Pharmacy specifies that more than 10,000 Internet websites selling medicines do not adhere to the pharmacy and practice standards [20].

Counterfeit products encountered on the Internet comprise a diverse range of pharmacological classes and formulations. Thus, Interpol carries out yearly operations in order to tackle the sales of counterfeit medicines online [21]. In 2015, operation Pangea VIII involved the seizures of 20.7 million counterfeit and illicit medicines including antihypertensive medicines, anticancer agents, sexual stimulants, and nutrition supplements [21]. Additional counterfeit products found on the Internet in other studies included anabolic steroids, anticancer, antiviral, antidepressants, anxiolytics, contraceptive, sexual stimulants, and weight-loss medicines [19,20,22-24]. In this case, anabolic steroids, along with anxiolytics and weight-loss pills sold on the Internet, were seized in the United Kingdom [22]. Anticancer agents encountered in the United States were Avastin in 2012 [20]. Additionally, Tamiflu purchased online was shown to contain a mixture of paracetamol and talc instead of its API (oseltamivir) [19]. Also, a number of antidepressants/anxiolytics (ie, alprazolam, escitalopram, lorazepam, and zolpidem) purchased online were found to not contain their API and instead contain another antipsychotic (haloperidol) [19]. Moreover, contraceptive products purchased from the Internet under the brand “Ortho Eva” did not contain any API [23]. Counterfeit sexual stimulants sold on the Internet, mainly Viagra, have been reported in a number of studies [24]. Also, sibutramine was encountered instead of orlistat in counterfeit Xenical product [19].

The harm resulting from using counterfeit medicinal products could range from ineffectiveness to potentially lethal effects. It is noteworthy to mention that the harm resulting from counterfeit lifestyle products could be a great risk in both medicinal and non-medicinal products. For instance, toxic metals were detected in both counterfeit cosmetic and herbal products [25-27]. Nonetheless, the majority of the literature investigated harm associated with counterfeit medicinal products. Such harm was based on predicting the adverse effects of certain medicines or experience of severe toxicity/lethal effect with a counterfeit medicine. Only four studies in the literature evaluated pharmacist [28] and patient/consumer knowledge [4,6,29] of medicines, and/or herbal products and dietary supplements. However, these studies concentrated mainly on products used for medicinal purposes and did not evaluate additional lifestyle products (eg, cosmetics) used by consumers on a daily basis. Moreover, they did specify the degree of harm (ie, mild, moderate, or severe) resulting from the use of counterfeit lifestyle products.

Our work aimed to investigate consumer and patient attitudes toward the purchase of lifestyle products from the Internet, their knowledge about product effects, authenticity, and toxicity, as well as their experience with using counterfeit lifestyle products.

## Methods

### Study Design and Data Collection

A multinational Web-based study was designed in order to examine the knowledge and attitudes of patients and consumers toward lifestyle products sold on the Internet. Participants were eligible if they spoke English and purchased lifestyle products from the Internet. Participants who did not make any Internet purchases were excluded. Residents in 22 countries responded, including Belgium, Brazil, Canada, China, Finland, France, Germany, Hong Kong, Italy, Kuwait, Lebanon, Morocco, Pakistan, Palestine, Russia, Saudi Arabia, Thailand, Turkey, United Arab Emirates, United Kingdom, and United States.

Data were collected using an anonymous online questionnaire by Bristol Online Survey and were accessible for the lifetime of the questionnaire (ie, between May 2014 and May 2015). Participants worldwide were invited to complete the survey by sending the link through emails and personal communication. Also, the questionnaire was posted on social media websites and discussion forums (eg, Facebook and LinkedIn). The language of the questionnaire was English (see Multimedia Appendix 1).

The questionnaire was initially piloted at a local gym (n=15) and at the university (n=15) prior to the study in order to evaluate reliability and clarity of the information using Cronbach alpha. After 1 week, the questionnaire was retested for reliability with the same 30 participants. A few modifications were made based on the outcomes of the pilot study, and the final version of the questionnaire was used online to collect data.

### Questionnaire

The questionnaire was divided into six parts that covered the following areas (see Table 1): (1) demographics, (2) extent of buying lifestyle products from the Internet, (3) types of products purchased, (4) awareness of counterfeit products sold over the Internet, (5) experience with counterfeit lifestyle products, and (6) experience of harm (adverse effects) associated with the use of counterfeit or poor-quality lifestyle products (Multimedia Appendix 1).

**Table 1:** Areas covered by the questionnaire.

Part	Aims
Demography	Sociodemographic
	Economic
Extent of buying products from the Internet	Frequency of buying products
	Sources of products
	Details of online retailers/pharmacies
Types of the products purchased	Medicinal products
	Non-medicinal products (herbal, supplements, and cosmetics)
	Classes of products (image improvement or performance enhancement)
Awareness of counterfeit products sold over the Internet	Knowledge on counterfeit products
	Sources of knowledge
Experience with counterfeit lifestyle products	Experience with adverse effects
	Types of adverse effects
	Degree of harm

### Ethics

Ethical approval for the study was sought by Bournemouth University internal ethics committee. Moreover, respondents gave informed consent of their willingness to take part in this study at the introduction of the questionnaire. The introduction further clarified to the respondents that they could withdraw anytime from the study (Multimedia Appendix 1). Moreover, all respondents’ data were handled and stored anonymously.

### Definitions

Lifestyle products are those intended to enhance the physical appearance and/or physical/mental performance of individuals [1]. A counterfeit mark is defined as “a spurious mark which is identical with or is substantially indistinguishable from a registered mark” [30]. A counterfeit medicinal product is defined as medicine that is “fraudulently and deliberately mislabeled according to identity and/or source” [31]. A counterfeit medicinal product could contain no API, wrong API, wrong ingredients, or even defective packaging [31]. A cosmetic product is [32]:

Any substance or preparation intended to be placed in contact with various external parts of the human body (epidermis, hair system, nails, lips and external genital organs) or with the teeth and the mucous membranes of the oral cavity with a view exclusively or mainly to cleaning them, perfuming them, changing their appearance and/or correcting body odors and/or protecting them or keeping them in good condition.

### Data Analysis

Data analysis was conducted using SPSS v21 where descriptive statistics were applied in order to gather responses and explore outcomes. Moreover, responses from open-ended questions were investigated individually in relation to consumer and patient knowledge and attitudes toward lifestyle products, their authenticity, and the associated toxicity with counterfeit lifestyle products. As most data obtained were categorical variables, they were reported as numbers and frequencies.

## Results

The questionnaire yielded 320 respondents from different sources. Participants were invited to complete the questionnaire online. The responses were received via the website, and all were complete and usable.

### Sociodemographics

Information from 320 respondents was analyzed. The respondents included 91 females (28.4%), 227 males (70.9%), and 2 participants who did not disclose their gender (see Table 2). The majority (62.5%, 200/320) of the respondents were in the 18-25 years group, 47 (14.7%) in the 26-33 years group, and 43 (13.4%) in the 34-41 years group. The remaining age groups were represented in less than 10% of the patients. Most of the respondents were British (78.1%, 250/320), followed by Europeans (8.8%, 28/320), Asian (7.5%, 24/320), African (1.3%, 4/320), Australians (0.9%, 3/320), and Americans (0.63%, 2/320). In addition, the majority of the respondents were residents of the United Kingdom (85.0%, 272/320), Asia (6.3%, 20/320), and Europe (3.4%, 11/320). The educational level among the respondents was mainly at a higher degree level or above; 149 (46.6%) of the respondents had at least a Bachelor’s degree. In relation to the number of languages spoken among respondents, 88.4% (283/320) were monolingual and spoke English only. The remaining respondents were bilingual or trilingual and spoke the following languages in addition to English: Arabic, Danish, Dutch, French, German, Greek, Italian, Mandarin, Nepalese, Russian, Somali, Spanish, Swedish, Thai, Turkish, and Urdu.

**Table 2:** Sociodemographic characteristics of participants (N=320).

Parameter		n	Frequency, %
**Age group**			
	18-25	200	62.5
	26-33	47	14.7
	34-41	29	9.06
	42+	43	13.4
	Prefer not to say	1	0.31
**Gender**			
	Male	91	28.4
	Female	227	70.9
	Prefer not to say	2	0.63
**Nationality**			
	Africa (Ivorian, Moroccan, Nigerian, South African)	4	1.25
	Asia (Bangladeshi, Egyptian, Indian, Kuwaiti, Lebanese, Nepal, Palestinian, Pakistani, Russian, Saudi Arabian, Syrian, Thai, and Turkish)	24	7.5
	Australian	3	0.94
	Europe (British)	250	78.1
	Europe other (Belgian, Bulgarian, Columbian, Cypriot, Czech, Danish, Dutch, Finnish, French, German, Greek, Italian, Norwegian, Polish, Romanian, Spanish, Swedish)	28	8.75
	North America (American and Canada)	8	2.50
	South America (Brazilian, Colombian)	2	0.63
**Country of residence**			
	Africa	0	0
	Asia (China, Hong Kong, Kuwait, Lebanon, Morocco, Thailand, Turkey, Pakistan, Palestine, Russia, Saudi Arabia, United Arab Emirates)	20	6.25
	Australia	3	0.94
	Europe (UK)	272	85
	Europe (Belgium, Finland, France, Germany, Italy)	11	3.4
	North America (America and Canada)	13	4.06
	South America (Brazil)	1	0.31
**Second language (additional to English)**			
	None	283	88.4
	One second language (Arabic, Danish, Dutch, French, German, Greek, Italian, Nepalese, Russian, Somali, Spanish, Swedish, Thai, Turkish, Urdu)	17	5.31
**Education level**			
	School/College	109	34.1
	Bachelor’s degree	149	46.6
	Master or post graduate	44	13.8
	PhD+	18	5.63

### Consumer and Patient Attitude Toward the Purchase of Lifestyle Products

According to the survey, 208 (65.0%) of the respondents reported purchasing lifestyle products from the Internet (see Table 3). However, only 17 (5.3%) claimed to have purchased lifestyle products frequently, whereas 111 (34.7%) purchased products occasionally and 91 (28.4%) rarely purchased these products. The main websites used for purchase of products were Amazon (64.1%, 205/320), eBay (39.7%, 127/320), online retailers’ websites (32.8%, 105/320), and online pharmacies (14.7%, 47/320). On the other hand, a very small proportion (<10%) used a “drug” website or Alibaba. Retailers websites reported included (1) cosmetic retailers: All Beauty, Alvin Connor, Bodyshop, Dermashop, MAC make up, (2) health care and beauty online retailers: Boots, Healthspan, Holland and Barrett, Love Melatonan, My Protein, Superdrug, (3) online department stores: Debenhams, Feel Unique, John Lewis, QVC, (4) supermarkets, (5) other websites such as Groupon, and (6) wholesale South Asian suppliers. When asked whether the country of the website was identifiable, fewer than half (44.7%, 143/320) of respondents could identify the countries of origin of the websites, which were France, Germany, India, Japan, United Kingdom, and United States. Some respondents claimed they could identify countries of origin of online pharmacies, which were mainly the United Kingdom (23.8%, 76/320) and United States (5.3%, 17/320). Other countries reported as sources for online pharmacies were Australia, Canada, China, Czech Republic, Finland, Germany, Greece, Holland, India, Pakistan, Philippine, Singapore, Sweden, Switzerland, and Thailand. Among the respondents who already bought medicines from UK online pharmacies, only 53 (16.6%) could recognize the Medicine and Healthcare Regulatory Agency (MHRA) logo. Moreover, only 17 (5.3%, 17/320) of respondents had consultations with a doctor at the online pharmacy. Subsequently, the sources of information obtained by the respondents regarding their products were mainly from the Internet (45.3%, 145/320) or family/friends (43.4%, 139/320) (see Table 3). Internet sources reported to be used by respondents were product websites, blogs/forums/chat rooms, YouTube, scientific papers, Facebook, and NHS (National Health Service) Direct website. In addition, a lower percentage of respondents obtained information on lifestyle products from interacting with health care professionals (24.1%, 77/320), magazines (17.8%, 57/320), and TV (13.1%, 42/320).

**Table 3:** Consumer and patient attitudes toward purchasing lifestyle products from the Internet.

Criteria		n	Frequency, %
**Purchase of lifestyle products over the Internet**			
	Yes	208	65
	No	112	35
**Frequency of purchase**			
	Always	17	5.31
	Occasionally	111	34.7
	Rarely	91	28.4
	Never	101	31.6
**Websites mostly used**			
	Alibaba	7	2.19
	Amazon	205	64.1
	eBay	127	39.7
	Drugs websites	21	6.56
	Online pharmacies	47	14.7
	Others (All Beauty, Alvin connor, Beauty base, Bodyshop, Boots, Debenham, Dermashop, Feel unique, Groupon, Healthspan, Holland and Barrett, John Lewis, Love Melanotan, MAC makeup, My protein,QVC, Superdrug, Tesco, Wholesale Indian suppliers)	105	32.8
**Country of the website identifiable**			
	Yes (France, Germany, India, Japan, UK, and US)	143	44.7
	No	103	32.2
	N/A	74	23.1
**Country of origin for medicines bought from online pharmacies**			
	UK	76	23.8
	USA	17	5.31
	India	6	1.88
	Canada	4	1.25
	Germany	4	1.25
	Australia	2	0.63
	Holland	2	0.63
	Other countries one pharmacy in each (China, Czech Republic, Finland, Greece, Pakistan, Philippines, Singapore, Sweden, Switzerland, Thailand)	10	3.13
**MHRA logo for authenticity for UK online pharmacies**			
	Yes	53	16.6
	No	30	9.38
	N/A	237	74.1
**Consultation with a doctor at the online pharmacy**			
	Yes	17	5.31
	No	115	35.9
	N/A	188	58.8
**Sources of information about lifestyle products**			
	Family/friends	139	43.4
	Health care professionals	77	24.1
	Magazines	57	17.8
	TV	42	13.1
	Others (websites, forums/blogs, Internet ads, smartphone apps, YouTube, Scientific papers, Product information leaflet, Facebook, NHS Direct)	145	45.3

### Types of Lifestyle Products Purchased

The results of the questionnaire showed that the main lifestyle products purchased from the Internet were cosmetic products (55.6%, 178/320) representing more than half of the total products (see Table 4). This was followed by supplementary/nutritional lifestyle products, drugs and herbal products, which represented 28.8% (92/320) and 18.8% (60/320) of the total purchased products respectively. Medicinal products represented the lowest percentage of the purchased lifestyle products (11.9%, 38/320).

Half the respondents 171 (53.4%) claimed that the lifestyle products they purchased from the Internet were available in stores in main shopping areas. They gave several reasons that made them purchase products online. The main justifications were convenience (25.9%, 83/320), lower cost/better offers (25.9%, 83/320), and easier alternative to in-store purchase (15%, 48/320). However, very few (4.7%, 15/320) participants believed Internet purchases were timesaving and quick, and only 6 (1.9%, 6/320) said that Internet purchases saved them from the embarrassment that could be encountered in stores when buying personal products. Also, less than 1% of respondents reported that Internet purchases provided a wider variety of products and offered more details on the products than those given by the pharmacist/store assistant. Even fewer reported that they favored Internet purchases because of better-quality products and ability to buy prescription medicines without a prescription.

Cosmetic products used for skin and physical appearance were the most commonly purchased products and were used by more than 60% of respondents. This was followed by lifestyle products used for mood and social behavior and those used for cognitive function, which were used by 34.7% (111/320) and 29.1% (93/320) respondents. Weight loss products and sexual stimulants were used by 20-30% of the respondents, while muscle enhancers were used by 16.3% (52/320) of respondents.

More specifically, the subclasses reported in each product category varied between each category. For cosmetic products, the majority of products used were hair products (n=220), acne products (n=149), moisturizers (n=140), sunscreens (n=55), and tanning solutions (n=43). In the remaining categories, herbal products formed the main category purchased. Products purchased in relation to mood and social behavior included mainly herbal sleep aids (n=50), mood enhancers (n=19), stimulants (n=19), and antidepressants (n=18). Cognitive enhancers were mainly caffeine (n=62) and natural memory enhancers (n=20). Only 7 respondents claimed the use of nootropics (synthetic medicines) for cognitive function. Similarly, synthetic medicines were underrepresented in the sexual stimulant category. Thus, the main purchases in this category were condom-type products, whereas only 14 (4.4%) respondents reported the purchase of Viagra, Cialis, and Levitra tablets. Herbal weight loss products (n=42) were preferred over other synthetic appetite suppressants (n=20), fat binders (n=16), or meal replacements (n=6). Furthermore, proteins (n=49) were favored over steroids (n=3) for muscle enhancers.

**Table 4:** Types of lifestyle products purchased from the Internet.

Criteria		n	Frequency, %
**Type of products purchased online**			
	Cosmetic	178	55.6
	Herbal	60	18.8
	Medicinal	38	11.9
	Supplementary and nutrition	92	28.8
	Others (drugs/legal highs, essential oils, condoms)	88	27.5
**Availability of products ordered online in community pharmacies/stores**			
	Yes	171	53.4
	No	149	46.6
**Reasons for buying products online**			
	Quick/time saving	15	4.69
	Cheap	83	25.9
	Easy	48	15
	Convenient	83	25.9
	More details on product than ones provided by the pharmacist	2	0.63
	No need for embarrassment of communicating with pharmacist	6	1.88
	Lack of availability of products in stores	9	2.81
	Not able to get prescription for some products	3	0.94
	Wide variety of products and offers	2	0.63
	Ability to purchase illegal drugs	1	0.31
	Better quality products	1	0.31
**Cognitive function**			
	Caffeine	62	19.4
	Natural memory enhancers	20	6.25
	Nootropics	7	2.19
	Others (multivitamins, cod liver oil, stimulant drugs)	4	1.25
	Total	93	29.1
**Mood and social behavior**			
	Antidepressants	18	5.63
	Mood enhancers	19	5.94
	Sleep aids	50	15.63
	Highs	19	5.94
	Others (psychedelics, anxiolytics, addiction management, pain management)	5	1.56
	Total	111	34.7
**Physical appearance**			
	Acne products		
	Creams/ointments/gels	95	29.7
	Solutions	12	3.75
	Tablets	42	13.1
	Hair products		
	Hair loss	9	2.81
	Cream/gel	48	15
	Shampoo	96	30
	Tablet	17	5.31
	Hair dye	50	15.6
	Other (nail polish, mascara, deodorant, perfume, lip balm, herbal products, protein shapes, make-up, shampoo)	17	5.31
	Total	237	74.1
**Muscle enhancers**			
	Proteins	49	15.3
	Steroids	3	0.94
	Total	52	16.3
**Skin products**			
	Moisturizers	140	43.8
	Skin lighteners	10	3.13
	Sunscreens	55	17.2
	Tanning solutions	43	13.4
	Other (foundation, body scrub, body oil)	5	1.56
	Total	253	79.1
**Weight-loss products**			
	Appetite suppressants	20	6.25
	Fat binders	16	5
	Herbal products	42	13.1
	Other (juices and meal replacement, diet shakes, diet pill)	6	1.88
	Total	84	26.3
**Sexual stimulants**			
	Condom products	44	13.8
	Herbal products	12	3.75
	Libido enhancers	6	1.88
	Viagra	7	2.19
	Cialis	3	0.94
	Levitra	4	1.25
	Total	76	23.8

### Awareness of Counterfeit Lifestyle Products Sold Via the Internet

**Figure 1:**
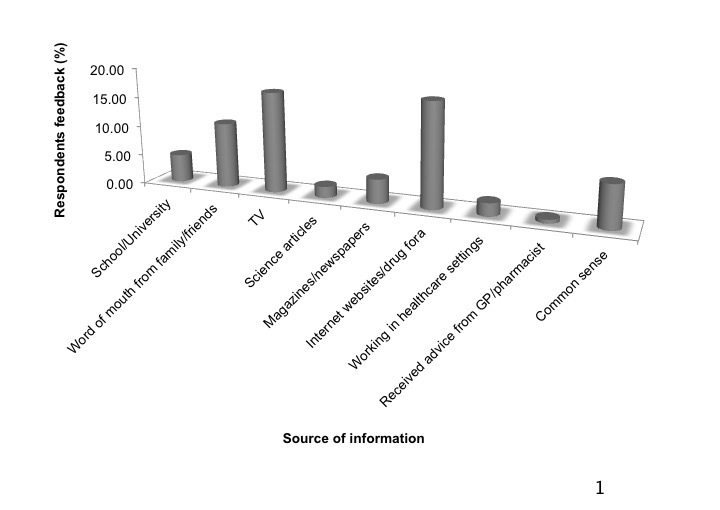
Sources of information obtained by consumers regarding counterfeit lifestyle products.

Most respondents (62.8%, 201/320) were aware of counterfeit lifestyle products being sold via the Internet. However, the sources of information regarding product counterfeiting varied between respondents (see Figure 1). In the majority of cases, respondents relied on information from Internet websites/drug forums (17.5%, 56/320), TV (16.9%, 54/320), and family/friends (10.9%, 35/320). Also, 25 (7.2%) respondents claimed that awareness of counterfeits is “common sense.” Moreover, 13 (4.1%) respondents gained knowledge about counterfeiting from magazines and newspapers. Furthermore, 7 (2.2%) respondents knew about counterfeiting from their job within a health care setting. Education was not a major source, as 15 (4.7%) respondents claimed they learned about counterfeiting in school/university degree and only 6 (2.0%) read scientific articles on the topic. Additionally, advice received from health care professionals was not enough regarding counterfeiting as only 2 respondents reported that their general practitioner and pharmacist explained product counterfeiting to them.

### Experience of Counterfeit Lifestyle Products

When asked about experience with a counterfeit product, 38 (11.9%) respondents reported having experience with a counterfeit lifestyle product (see Table 5). In only 3 cases was this product reported to an authority. Moreover, when asked how they could identify a counterfeit product, respondents claimed that they would look at the product’s packaging (12.8%. 41/320), appearance (11.6%, 37/320), or label claim (5.6%, 18/320). Fewer respondents stated that they would know from the product’s efficacy (2.5%, 8/320) or side effects (4.1%, 13/320).

Respondents were aware of the risks associated with the counterfeit products. However, 45 (14.1%) respondents said that it was acceptable to take the risk of buying potential counterfeit products in case of emergencies such as medicine shortage or poor finance. They further clarified that it is up to the individual who is aware of the problem to make the decision especially in case of poor finance, lack of availability in online stores, or “if the product is not a medicine.”

**Table 5:** Consumer experience with counterfeit lifestyle products.

Criteria		n	Frequency, %
**Experience of a counterfeit products**			
	Yes	38	11.9
	No	282	88.1
**Identification of a counterfeit product**			
	Different appearance	41	12.8
	Different packaging	37	11.6
	No label	18	5.63
	No packaging	10	3.13
	Side effects	13	4.06
	Wrong ingredients	8	2.50
	Other (not effective, different brand printed on the label, adverse effects not stated on the product information leaflet, cosmetics giving lighter colors)	7	2.19
**Was the counterfeit product reported to the authority**			
	Yes	3	0.94
	No	99	30.9
	Other (reported to eBay)	1	0.31
**Risking buying a potential counterfeit product from the Internet due to medicine shortage or lower prices**			
	Yes	45	14.1
	No	249	77.8
	Not applicable	26	8.13
**Reasons for risking buying a potential counterfeit product from the Internet due to medicine shortage or lower prices**			
	Cheaper	7	2.19
	Easier	3	0.94
	Lack of availability in stores	4	1.25
	Flexibility	1	0.31
	Up to individual after becoming aware of the problem	4	1.25
	Depends on the product (okay for cosmetics but not for drugs)	4	1.25

### Experience of Adverse Effects Resulting From Counterfeit Lifestyle Products

Adverse effects resulting from the use of counterfeit products were below 10% (see Table 6); only 23 (7.2%) respondents claimed they had adverse effects resulting from a counterfeit product purchased from the Internet. Of these, 17 reported that the adverse effects were not stated on the product information leaflet and were instead due to the poor quality. The types of adverse effects varied between products and were mainly encountered with cosmetic and herbal products. Skin reactions (eg, allergy, rash, itching, swelling) as well as eye infection were experienced with counterfeit cosmetic products. In addition, increased blood pressure/heart rate, appetite suppression, urinary tract infection, and gastrointestinal disturbances were reported with herbal products. Only 4 respondents reported these adverse effects to the authorities/source of purchase, and only 1 respondent received treatment for these adverse effects. However, about half of respondents (49.4%, 158/320) believed that the extent of harm resulting from counterfeit products could be lethal.

**Table 6:** Consumers’ experience with adverse reactions associated with the use of counterfeit lifestyle products.

Criteria			n		Frequency, %
**Experience of an adverse effect from products purchased online**					
	Yes	23		7.19	
	No	297		92.81	
**Were the side effects stated in the products’ information leaflet**					
	Yes	17		5.31	
	No	258		80.6	
	No label	45		14.1	
**Types of adverse effects experienced**					
	Skin reactions (allergy, rash, itching, swelling)	15		4.69	
	Increased blood pressure	2		0.63	
	Increased heart rate	2		0.63	
	Eye infection	1		0.31	
	Urinary tract infection	1		0.31	
	Nausea, vomiting, gastrointestinal disturbances	8		2.5	
	Appetite suppression	1		0.31	
**Reported adverse effects**					
	Yes	4		1.25	
	No	143		44.7	
**Receipt of treatment for adverse effects**					
	Yes	1		0.31	
	No	22		6.88	
**Extent of harm resulting from counterfeit product**					
	Very mild	15		4.69	
	Mild	33		10.3	
	Average	31		9.69	
	Harmful	83		25.9	
	Lethal	158		49.4	

## Discussion

### Principal Findings

This study examined consumer and patient attitudes toward the purchase of lifestyle products from the Internet. Although previous literature surveys examined the attitudes of patients towards online pharmacies [4,6,29], they did not evaluate products such as cosmetics and herbal medicines that could impact public health or patient safety. To our knowledge, this was the first study to address user perceptions regarding counterfeit lifestyle products. Lifestyle products have witnessed a global increase in recent years due to the change in people’s attitude, way of living [2], and the introduction of personalized care [1,11]. Personalized care consists of licensed/unlicensed products that focus mainly on improving an individual’s performance, image, mood, appetite, sleep, and sexual desires [1]. Moreover, these products were found to be sold on nearly every website in a study evaluating 136 online pharmacies [11].

The research showed that more than half of respondents purchased lifestyle products from the Internet. This was much higher than previous investigations that reported 8.3% [6], 14.5% [29], and 16% [4] of consumers bought medicinal products from the Internet. This could be attributed to the fact that the latter investigations evaluated only medicines, whereas our study included medicines, cosmetics, and herbal products. This would imply that additional lifestyle products were underestimated in previous studies despite their impact on public health. Moreover, the aforementioned surveys were limited to participants from a small range of countries, whereas this survey had respondents of 40 nationalities from 22 countries.

The majority of participants spoke only English, and a minority of them spoke two or more languages. The majority of the participants were UK residents, which influenced the types of lifestyle products purchased. Amazon and eBay were the most popular sites mentioned by respondents. This was followed by known health care and beauty retailer websites such as Boots and Superdrug. In contrary to other studies [4], online pharmacies were used by only 14% of participants. Less than half of participants were aware of the country of origin of the website/online pharmacy they were buying from. Specifically for online pharmacies, only 16% could recognize the MHRA logo for licensed online pharmacies. Also, respondents did not communicate with cyber doctors for information on lifestyle products. However, they did count mainly on family and friends, the media, or the Internet. Only a quarter of respondents obtained information from health care professionals. This was also noted in other studies that showed that the lack of communication between health care professionals and patients affected the desired treatment outcome [33].

It is likely that respondents did not consult with health care professionals because more than half of the products purchased from the Internet were cosmetics. Though the products were available in stores, respondents preferred purchasing them from the Internet as it offered quicker, cheaper, and more convenient alternatives to stores [4,33,34]. The cosmetics were mainly hair/face products, acne products, and tanning solutions. Additionally, respondents used products that improved their mental performance (eg, mood, social behavior, and cognitive function) and physical appearance (eg, weight loss products, sexual stimulants, and muscle enhancers).

The survey found that more than half of respondents were aware of counterfeit products. Respondents referred to Internet websites, media, and family/friends as the major source of information on counterfeit products; however, half of them did not check the origin of the website when purchasing lifestyle products. This observation was in agreement with other surveys that showed that consumers relied on information obtained from the Internet regarding their products [35,36].

In only 1% of cases were counterfeit products reported to the authorities or the supplier. Some respondents did not report counterfeit products and underestimated their dangerous consequences. They believed it acceptable to risk buying counterfeit products in the case of medicine shortage, poor finance, lack of product availability in stores, or in cases of non-medicinal products. This was because of the perception that non-medicinal products (such as cosmetics) were considered less dangerous and harmful to the consumer.

The adverse effects experienced from the use of lifestyle products in this survey were relatively low (<10%). In most cases, adverse effects were attributed to the product itself and corresponded to effects described in the patient information leaflet (PIL). On the contrary, adverse effects not stated on the PIL were attributed to the product being potentially counterfeit and included skin reactions (eg, allergy, rash, itching, and swelling), cardiovascular effects (eg, increased heart rate and blood pressure), gastrointestinal effects (eg, nausea, vomiting, and gastrointestinal disturbance), eye infection, and urinary tract infection. Yet in only 1% of the cases were these adverse effects reported to authorities or a treatment sought. However, more than three-quarters of participants believed that counterfeit medicines could be harmful or lethal.

### Limitations

The first limitation in this study was our sample size of 320 participants. The low response rate could be attributed to the complexity of the survey and can be improved with further development and testing. More specifically, the study sample size was low in some countries including Australia, China, Czech Republic, Finland, Greece, Holland, Pakistan, Philippine, Singapore, Sweden, Switzerland, and Thailand. The majority of responses were from the United Kingdom. In this respect, caution should be taken in interpreting the generalizability of the findings in the aforementioned countries. Moreover, the questionnaire was self-reported; thus, there could be potential information bias. Furthermore, the research is not itself conclusive and more research is needed to explore the association between counterfeit products and side effects experienced.

### Conclusions

The results of this study showed that more than half the respondents purchased lifestyle products from the Internet. The majority of the respondents purchased cosmetics; whereas, only a small minority purchased medicines from the Internet. The main reasons attributed to purchasing lifestyle products from the Internet were convenience, low prices, detailed product information, and consumer privacy. Most respondents were aware of the presence of counterfeit lifestyle products on the Internet. The main source of information about counterfeit products was obtained through media and/or family and friends. However, only 11.9% of patients experienced counterfeit lifestyle products, of whom only 1% reported it to authorities. Only 7.2% of patients experienced adverse effects associated with the use of a counterfeit lifestyle product. Nonetheless, all respondents were aware of the dangers associated with the use of counterfeit lifestyle products. However, 14.1% of respondents considered it acceptable to risk buying counterfeit products in the case of poor finance, lack of availability in store, or for non-medicinal products.

## Appendix

Multimedia Appendix 1 Online survey questionnaire.

## Abbreviations

API: active pharmaceutical ingredient
FDA: Food and Drug Administration
MHRA: Medicine and Healthcare Regulatory Agency
NHS: National Health Service
PIL: patient information leaflet
