# Supplementary material for: Exploring Consumer and Patient Knowledge, Behavior, and Attitude Toward Medicinal and Lifestyle Products Purchased From the Internet: A Web-Based Survey
Source: JMIR Public Health Surveill. 2016 Jul 18;2(2):e34. doi: 10.2196/publichealth.5390 (PMC4969549; doi:10.2196/publichealth.5390)
Supplement: Multimedia Appendix 1 [file publichealth_v2i2e34_app1.pdf]

Introduction

Counterfeit lifestyle products represent a significant health and public concern; especially if they subsequently lead to serious health consequences. Lifestyle products are one of the most frequently bought items on the Internet. However, the extent of purchasing these products online and its level of harm on consumers are still under-researched worldwide and specifically in the UK.

Therefore, the aim of this questionnaire is to investigate the extent of purchasing lifestyle products from the Internet. In addition, the questionnaire aims to examine the participants' awareness of the potential hazards which could happen due to uncontrolled use of online pharmacies and products purchased via them.

Your participation is entirely voluntary. There is no right or wrong answer and I am interested in your own personal experiences and point of views. The identities of all participants will remain strictly confidential and it will not be possible to identify any individual. The overall aim of the study is to make recommendations to reduce the risks of uncontrolled use of online pharmacies. This questionnaire should take around 15 minutes.

Part I: Demography

Please select your age range

- [ ] 18-25
- [ ] 26-33
- [ ] 34-41
- [ ] 42+

What is your gender?

- [ ] Male
- [ ] Female

What is your nationality?

[ ]

Which country do you reside in?

[ ]

What language(s) do you speak?

[ ]

What is your education level?

- [ ] School / College
- [ ] University Bachelors Degree
- [ ] Masters or other Post Graduate
- [ ] PhD +

Part II: Extent of Buying Lifestyle Products Online

Do you purchase lifestyle products over the Internet?

- [ ] Yes
- [ ] No

If yes, how often do you purchase them?

- [ ] Rarely
- [ ] Occasionally
- [ ] Always

What websites do you use?

- [ ] Alibaba
- [ ] Amazon
- [ ] eBay
- [ ] Drugs Websites
- [ ] Online Pharmacies
- [ ] Other (Please Specify):

[ ]

Is the country of origin of the <u>website</u> identifiable?

- [ ] Yes (Please Specify)
- [ ] No

[ ]

If you buy from <u>online pharmacies</u>, what country do they originate?

[ ]

For UK online pharmacies, do they have this badge?

Registered Pharmacy 1108466

- [ ] Yes
- [ ] No

Do you speak to a doctor online to seek advice before you buy them?

- [ ] Yes
- [ ] No

From which sources do you get the information about these products?

- ☐ Healthcare Professionals
- ☐ TV
- ☐ Magazines
- ☐ Family/Friends
- ☐ Other (Please Specify):

Part III: What Do You Purchase?

What types of products do you purchase online?

- ☐ Medicinal Products
- ☐ Herbal Products
- ☐ Supplementary and Nutritional Products
- ☐ Other (Please Specify):

Are the products you purchase online available in community pharmacies?

- ☐ Yes
- ☐ No

If yes, why do you purchase them online?

Which of these have you purchased?

Cognitive Function:

- ☐ Caffeine
- ☐ Natural Memory Enhancers
- ☐ Nootropics
- ☐ Other (Please Specify):

Weight Loss:

- ☐ Appetite Suppressants
- ☐ Fat Binders

Mood and Social Behaviour:

- ☐ Antidepressants
- ☐ Mood Enhancers
- ☐ Sleep Aid
- ☐ Too Get High
- ☐ Other (Please Specify):

Physical Appearance:

- ☐ Acne Products (If yes, Please Specify):
  - ☐ Cream/Ointment/Gel
  - ☐ Solution
  - ☐ Tablet
- ☐ Hair Products (If yes, Please Specify):
  - ☐ Hair Loss:
    - ☐ Cream
    - ☐ Shampoo
    - ☐ Tablet
    - ☐ Other (Please Specify):
  - ☐ Hair Dye
  - ☐ Other (Please Specify):

Muscle Enhancers:

- ☐ Proteins
- ☐ Steroids
- ☐ Other (Please Specify):

Skin Products:

- ☐ Moisturisers
- ☐ Skin Lighteners
- ☐ Sun Screens
- ☐ Tanning Solutions
- ☐ Other (Please Specify):

Have you ever experienced a

- [ ] Herbal Products
- [ ] Other (Please Specify):

Sexual Stimulants:

- [ ] Condom Products
- [ ] Herbal Products
- [ ] Libido Enhancers
- [ ] Synthetic:
  - [ ] Viagra
  - [ ] Cialis
  - [ ] Levitra
- [ ] Other (Please Specify):

Part IV: Awareness of Counterfeit Products

Are you aware of counterfeit medicines on the Internet?

- [ ] Yes
- [ ] No

If yes, can you please tell what do you know about them and where did you get this information from?

If yes, how would you check the originality of online pharmacies?

- [ ] Official Registered Pharmacies
- [ ] Badge
- [ ] Food and Drug Administration (FDA)
- [ ] World Health Organisation (W.H.O)
- [ ] I do not know
- [ ] Other (Please Specify):

If yes, were the effects different to the possible side effects stated on the products label?

- [ ] Yes

counterfeit product?

- [ ] Yes
- [ ] No

If yes, how did you identify it?

- [ ] Different Appearance
- [ ] Different Packaging
- [ ] No Label
- [ ] No Packaging
- [ ] Side Effects
- [ ] Wrong Ingredient
- [ ] Other (Please Specify):

If yes, did you report this to an authority?

- [ ] Yes (Please Specify)
- [ ] No (What did you do with the product?)

Part V: Awareness of Adverse Effects

Do you believe it is acceptable to take the risk of purchasing unofficial products (possibly counterfeit) on the Internet due to medicine shortage or lower prices?

- [ ] Yes (Why do you think it is worth doing?)
- [ ] No

Have you ever experienced any adverse reactions from a product purchased over the Internet?

- [ ] Yes
- [ ] No

☐ No<br>☐ No side effects stated on the label<br><br>What type of adverse effects have you experienced?<br>[ ]<br><br>**Did you report these effects to the website you purchased from, or any other authority?**<br>☐ Yes (Please Specify)<br>[ ]<br>☐ No<br><br>**Did you get any treatment or advice from health care professionals regarding these adverse effects? Please Specify:**<br>[ ]<br><br>**What do you believe is the extent of harm resulting from counterfeit products obtained from online sources on a scale of 1-10?** (1 - Very mild, 10 - Lethal):<br>☐ 1<br>☐ 2<br>☐ 3<br>☐ 4<br>☐ 5<br>☐ 6<br>☐ 7<br>☐ 8<br>☐ 9<br>☐ 10	
